# Decision-making in primary onset middle-age type 2 diabetes mellitus: a BOLD-fMRI study

**DOI:** 10.1038/s41598-017-10228-x

**Published:** 2017-08-31

**Authors:** Dan-Miao Sun, Ye Ma, Zong-Bo Sun, Lei Xie, Jin-Zhuang Huang, Wei-Song Chen, Shou-Xing Duan, Zhi-Rong Lin, Rui-Wei Guo, Hong-Bo Le, Wen-Can Xu, Shu-Hua Ma

**Affiliations:** 1 The First Affiliated Hospital of Shantou University Medical College, Shantou, Guangdong Province 515041 China; 2 Graduate School of Beijing Normal University, 519087 Zhuhai, China; 3 Guang dong Key Laboratory of Medical Molecular Imaging, 515041 Shantou, China

## Abstract

Although type 2 diabetes mellitus (T2DM) is a well-recognized risk factor for dementia, the neural mechanisms that underlying cognitive impairment in T2DM remain unclear. We used functional magnetic resonance imaging (fMRI) during a computerized version of the Iowa Gambling Task to investigate the neural basis of decision making at the initial onset stage of T2DM. Eighteen newly diagnosed middle-aged T2DM patients, with no previous diabetic treatment history, and 18 matched controls were recruited. Results indicated that T2DM patients made more disadvantageous decisions than controls. Compared to healthy subjects, T2DM patients showed decreased activation in the ventral medial prefrontal cortex (VMPFC), orbitofrontal cortex (OFC) and anterior cingulate cortex, and increased activity in the dorsolateral prefrontal cortex, posterior cingulate cortex, insula and occipital lobes. IGT performance positively correlated with changes in brain activation in the VMPFC and OFC in both groups. Moreover, poor glycemic control was associated with decision-making function both in behavioral and brain activity in the VMPFC and OFC in patients. Conclusively, T2DM patients may suffer from weaknesses in their prefrontal cortex functions that lead to poorer decision-making under ambiguity, at least as assessed by the IGT.

## Introduction

Type 2 diabetes mellitus (T2DM) is s a common metabolic disease characterized by hyperglycemia due to insulin resistance and relative insulin deficiency. T2DM is associated with pathological cognitive function and behavioral changes, detectable by imaging, electrophysiology, neural biochemical, and neural psychological tests, which are in all referred to as “diabetic encephalopathies”^1^. T2DM may accelerate the aging process of the brain, and is an independent risk factor for mild cognitive impairment (MCI) and dementia^2–4^. MCI is an intermediate state between normal cognition and dementia, and has been suggested to occur during the early stages of T2DM^5^, making early identification of brain function changes more important, especially for the prevention of T2DM-related MCI and dementia.

At its most basic level, decision making is the capacity to make the optimal selection in terms of rewarding or punishing outcomes between several alternative courses of action, under ambiguous or risky situations^6^. Decision-making can be assessed using the Iowa Gambling Task (IGT)^7^, a card game simulating real-life decision-making processes, and which has been widely used to evaluate decisions made under ambiguous conditions. In the IGT, rules for gains and losses are implicit and the subject must balance probabilistic outcomes and variable magnitudes of reward/punishment under unclear conditions. They not only have to consider the benefit of decisions for their current living situation, but also to anticipate the consequences of a decision in the near and far future. This type of decision-making is frequently encountered in daily life and is highly relevant for making self-advantageous decisions regarding health care, medical treatment and financial issues.

The mechanism underlying the IGT is theoretical, explained by the Somatic Marker Hypothesis proposed by Damasio^7, 8^. The hypothesis suggests that decision-making process may be biased by the emotional signals generated from the body when facing different options. There is a general agreement that decision-making relies on several processing steps that are supported by specific brain areas and neurotransmitter systems. According to prior study on neural correlates of decision-making in healthy subjects^9^, the commonly activated regions induced by the IGT include: (a) neural systems critical for processing emotion, namely, the insula, posterior cingulate cortex and amygdala; (b) neural systems critical for working memory, namely, the dorsolateral prefrontal cortex (DLPFC) and hippocampus, to provide online knowledge during the decision-making process; (c)neural systems critical for regulatory competence, namely, the orbitofrontal cortex (OFC) and the ventral medial prefrontal cortex (VMPFC), to interlink the previous two processes and have been described as neural “bridge” between emotion and cognition; (d) neural systems critical for behavioral decisions, namely, the anterior cingulate cortex (ACC)/supplementary motor area and ventral striatum.

The IGT offers clinical value when being used to detect decision-making impairments that are characteristic of patients with prefrontal cortex lesions^7, 10^. Decision-making evaluated by the IGT has been described in a variety of neurological disorders such as MCI^11^, Alzheimer’s disease^12–14^, type 1 diabetes mellitus^15^, Parkinson’s disease^16, 17^ and multiple sclerosis^18–20^. However, few studies have employed the IGT in functional magnetic resonance imaging (fMRI) investigations in neurological patients^16^, and no study to date has been published regarding decision making on the IGT in T2DM patients.

Notably, several studies have shown that T2DM and Alzheimer’s disease have remarkably similar neuropathology^21, 22^. Previous neuroimaging studies of T2DM patients show that brain atrophy appears in the hippocampus, amygdala, and prefrontal areas, and it frequently occurs in elderly patients with long duration of diabetes^23^. Cognitive impairment across multiple domains of cognition has been demonstrated in T2DM, and involve executive functioning, memory, attention, information processing and visuospatial abilities^24–27^, even in studies with middle-age T2DM patients^28–31^. In line with these cognitive dysfunctions, neuroimaging studies show the functional brain changes in early stage T2DM as well, including altered resting-state functional connectivity and task-related neuronal activity^32–35^. Taking all evidence into consideration, it can be assumed that decision processes become altered in T2DM patients as the disease progresses. We aimed to bridge the knowledge gap concerning decision-making and T2DM patients because decision-making is being increasingly recognized as an important component of the neuropsychological characterization of T2DM. Patients with T2DM are required to make strict daily decisions for optimal glycemic control. It is important to examine functional alterations that may precede clinically evident cognitive decline, so that preventative therapies can be implemented in middle age.

In the present study, our principal aim was to explore the decision-making impairment in primary onset middle-age T2DM patients using behavioral and blood oxygenation level-dependent functional magnetic resonance imaging (BOLD-fMRI). We predicted that decision-making would be impaired in patients with T2DM compared with healthy control subjects. Since fMRI has the advantage of being highly sensitive to early changes in the neural substrates supporting cognition before clinically significant cognitive impairment^36^, fMRI enables us to study possible alterations of the neural correlates associated with the decision-making. Another goal of this study was to explore the link between fMRI information and IGT performance and clinical data, using Pearson’s correlation analysis.

## Results

### Clinical and neuropsychological data

Results of the demographic, clinical characteristics and the neuropsychological test battery are summarized in Table 1. There were no significant differences between groups in age, gender, education level, body mass index (BMI), triglyceride, total cholesterol, and systolic/diastolic blood pressure. As expected, T2DM patients exhibited significantly elevated glycosylated hemoglobin (HbA1c) and fasting glucose level compared to controls (all P < 0.001). In terms of cognitive performance, the diabetic group showed slightly lower Mini-Mental State Exam (MMSE) scores than healthy controls (T2DM group: 27.67 ± 1.50; Healthy control: 28.94 ± 1.11, P < 0.05), but scores of both groups were within the normal range. Then, T2DM patients had poorer total scores for the Montreal Cognitive Assessment (MOCA) compared with the control group (T2DM group: 24.56 ± 2.06; Healthy control: 27.28 ± 1.70, P < 0.001). No significant differences between groups were observed for Hamilton Depression Scale (H﻿AMD) scores.

**Table 1:** Demographic, clinical characteristics and neuropsychological data.

Items	T2DM patients (n = 18)	Healthy controls (n = 18)	p-value
Age (years)	42.89 ± 7.01	40.33 ± 4.14	0.194
Gender (female: male)	9:9	9:9	1.000
Education (years)	11.56 ± 2.41	11.94 ± 2.12	0.611
BMI (kg/m^2^)	25.78 ± 1.98	24.67 ± 2.02	0.106
Systolic blood pressure (mmHg)	119.39 ± 9.27	120.44 ± 11.27	0.761
Diastolic blood pressure (mmHg)	81.33 ± 8.575	78.33 ± 7.79	0.28
Fasting glucose (mmol/L)	13.30 ± 3.56	4.86 ± 0.39	<0.001*
HbA1C (%)	12.93 ± 1.64	4.56 ± 0.72	<0.001*
Triglyceride (mmol/L)	1.16 ± 0.46	0.91 ± 0.37	0.087
Total cholesterol (mmol/L)	4.94 ± 0.72	4.50 ± 0.88	0.113
MMSE	27.67 ± 1.50	28.94 ± 1.11	**0.006** ^*^
MOCA	24.56 ± 2.06	27.28 ± 1.70	<0.001*
HAMD	1.28 ± 0.75	0.83 ± 0.79	0.092

Data are mean ± standard deviation. *P < 0.05 was considered significant. Abbreviations: MMSE, Mini- Mental State Exam; MOCA, Montreal Cognitive Assessment; HAMD, Hamilton Depression Scale.

### IGT performance

**Figure 1:**
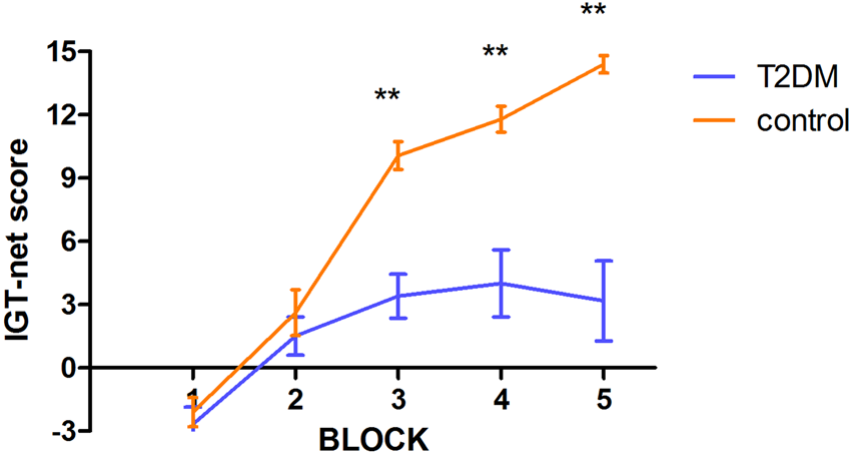
Performance of the IGT during 5 consecutive stages of the task. T2DM patients and healthy controls performed equally well in the first and second blocks (first 40 cards). From the third block, T2DM patients selected less advantageous decks than healthy controls and the differences in performance between groups became more pronounced as the task advanced. Positive scores reflected more advantageous choices, and negative scores reflected more disadvantageous choices. Error bars indicate the standard errors of the mean. **P < 0.001 was considered significant.

Figure 1 displays the change curve of the net scores. The total net score of the T2DM group in the IGT was significantly lower than that of the healthy control group (t = −5.362, p < 0.001). In a repeated-measures ANOVA performed on the IGT, results revealed a significant main effect for block (F = 57.437, p < 0.001) and for group (F = 28.750, p < 0.001), as well as for the interaction between group and block (F = 13.529, p < 0.001). Comparisons of the net score in each block showed significant differences between the two groups were detected in the third block (t = −5.047, P < 0.001), fourth block (t = −4.558, P < 0.001), and fifth block (t = −5.777, P < 0.001).

### Functional MRI data

**Figure 2:**
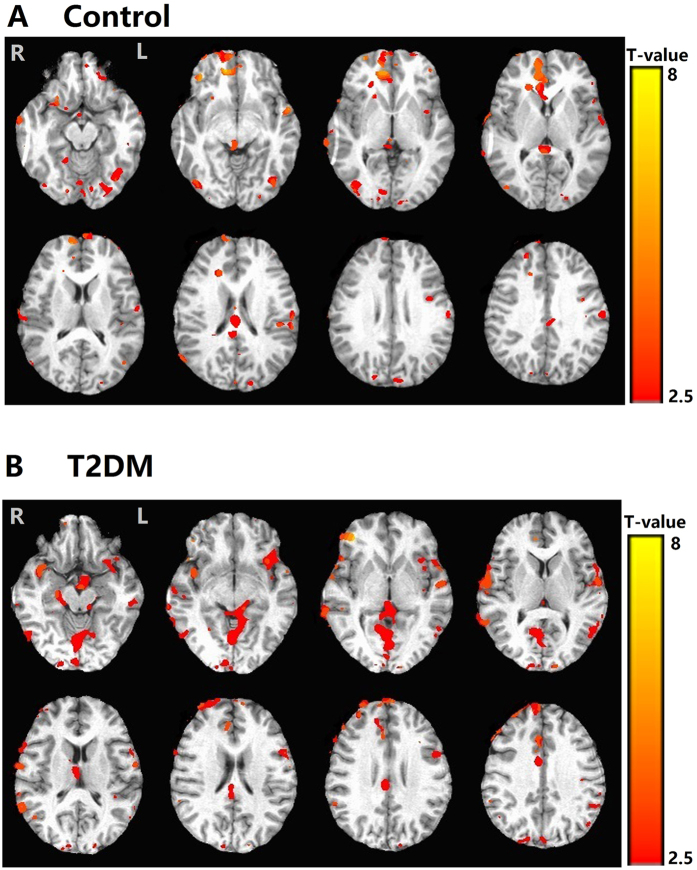
Brain activation during decision-making (decision- making - control conditions). (**A**) Brain activation during the Iowa Gambling Task compared to its control task in the control group. (**B**) Brain activation during the Iowa Gambling Task compared to its control task in the T2DM group. Common activated regions included the orbitofrontal cortex (OFC), ventral medial prefrontal cortex (VMPFC), anterior cingulate cortex (ACC), dorsolateral prefrontal cortex (DLPFC), insula, parietal and occipital lobes. Only healthy subjects showed activation within the ventral striatum and thalamus, only T2DM patients showed activation within the posterior cingulate cortex. See Table 2 for details.

The brain activity during decision-making versus control conditions showed similar activation patterns in both T2DM and control groups (Fig. 2, Table 2). Activated regions in common included the orbitofrontal cortex (OFC), ventral medial prefrontal cortex (VMPFC), anterior cingulate cortex (ACC), dorsolateral prefrontal cortex (DLPFC), insula, parietal and occipital lobes. However, only healthy subjects showed activation within the ventral striatum and thalamus, and only T2DM patients showed activation within the posterior cingulate cortex.

**Table 2:** Brain activation in the Control and T2DM group during decision-making (decision-making - control conditions).

Group	Anatomical region	R/L	Activation volume (mm³)	BA	Talairach coordinates (mm)			T value
					X	Y	Z	
Control	orbitofrontal	L	45	BA10	−8	45	−3	3.05
	ventral medial prefrontal cortex	L	30	BA47	−31	24	−8	2.83
		R	120	BA45	34	22	−4	6.57
	anterior cingulate cortex	L	21	BA32	29	−10	−10	2.56
		R	60	BA32	26	−19	38	3.85
	dorsolateral prefrontal cortex	L	28	BA46	29	−10	−10	2.77
		R	32	BA46	52	10	−22	2.94
	ventral striatum	R	22	—	7	1	44	2.60
	insula	L	38	—	−34	−25	−10	3.01
		R	32	—	32	−24	12	2.94
	parietal	L	28	BA7	53	−28	38	2.77
		R	40	BA7	52	−34	−23	3.15
	thalamus	L	24	—	11	−1	−1	2.67
	occipital	L	50	BA17	20	38	−3	3.65
		R	48	BA17	17	−58	44	3.58
T2DM	orbitofrontal	R	22	BA10	13	29	8	2.60
	ventral medial prefrontal cortex	L	20	BA45	−31	24	−8	2.50
		R	24	BA45	34	22	−4	2.67
	anterior cingulate cortex	R	32	BA32	29	−10	−10	2.94
	dorsolateral prefrontal cortex	R	78	BA46	52	10	−22	5.73
	insula	L	58	—	29	−10	−10	3.78
		R	68	—	32	−24	12	4.55
	posterior cingulate cortex	R/L	70	BA30	−22	−57	20	4.84
	parietal	L	44	BA7	53	−28	38	3.31
		R	40	BA7	52	−34	−23	3.15
	occipital	L	68	BA17	20	38	−3	3.01
		R	78	BA17	17	−58	44	5.01

P = 0.05; Cluster size = 20.L = left; R = right; BA = Brodmann area.

**Figure 3:**
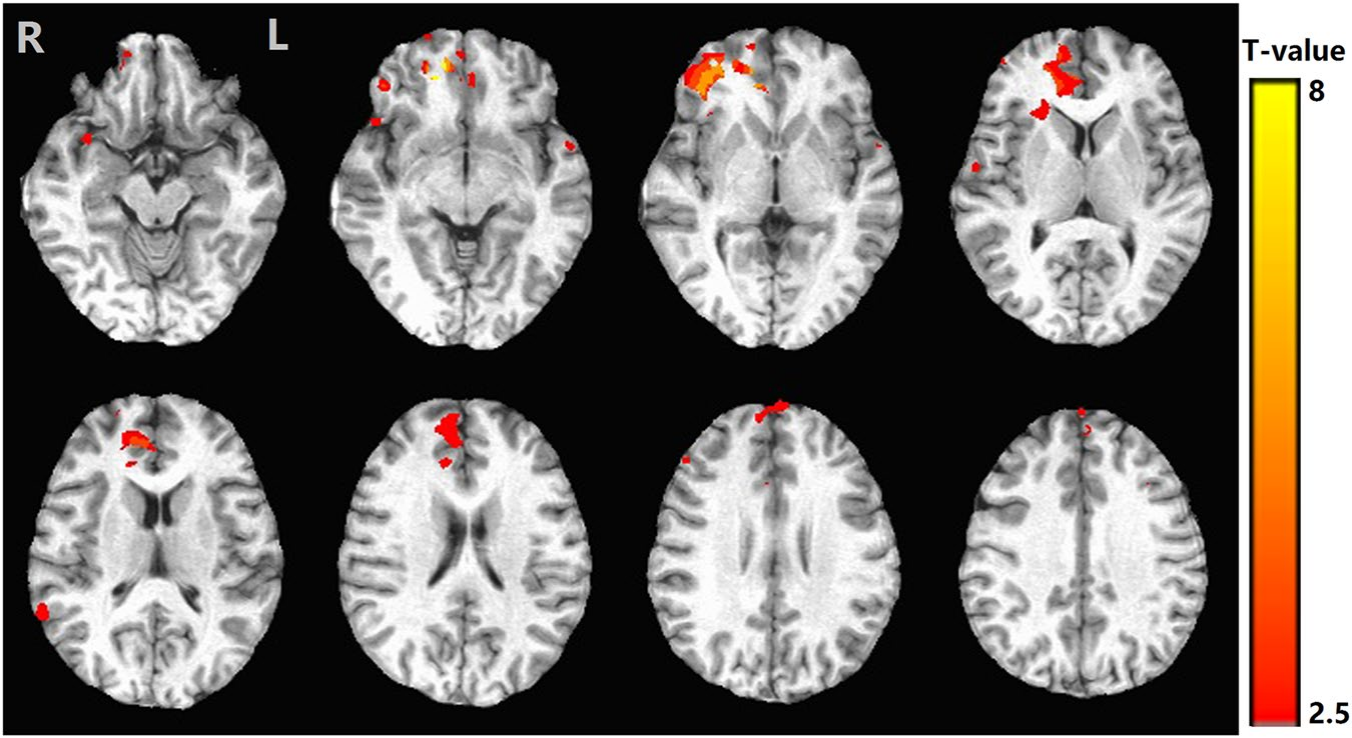
Group differences in brain activity during decision- making (Controls versus T2DM patients). As compared to T2DM patients, the controls showed increased activation in the orbitofrontal cortex (OFC), ventral medial prefrontal cortex (VMPFC) and the anterior cingulate cortex (ACC).

Statistical comparison of activation maps demonstrated that controls exhibited significantly greater activity in the OFC, VMPFC and ACC as compared to T2DM patients (Fig. 3). However, greater activity in T2DM patients, compared to controls, was observed in the right DLPFC, insula, posterior cingulate cortex and occipital lobes.

### **Correlational analysis data**

Correlations between performance and brain activity during the IGT were observed for the VMPFC and OFC. Results showed the activity in the VMPFC (r = 0.776, P < 0.001) and OFC (r = 0.487, P = 0.040) positively correlated with performance in controls. Positive correlations between brain activations in the VMPFC (r = 0.610, P = 0.007) and OFC (r = 0.480, P = 0.044) and net scores were also found in patients.

**Figure 4:**
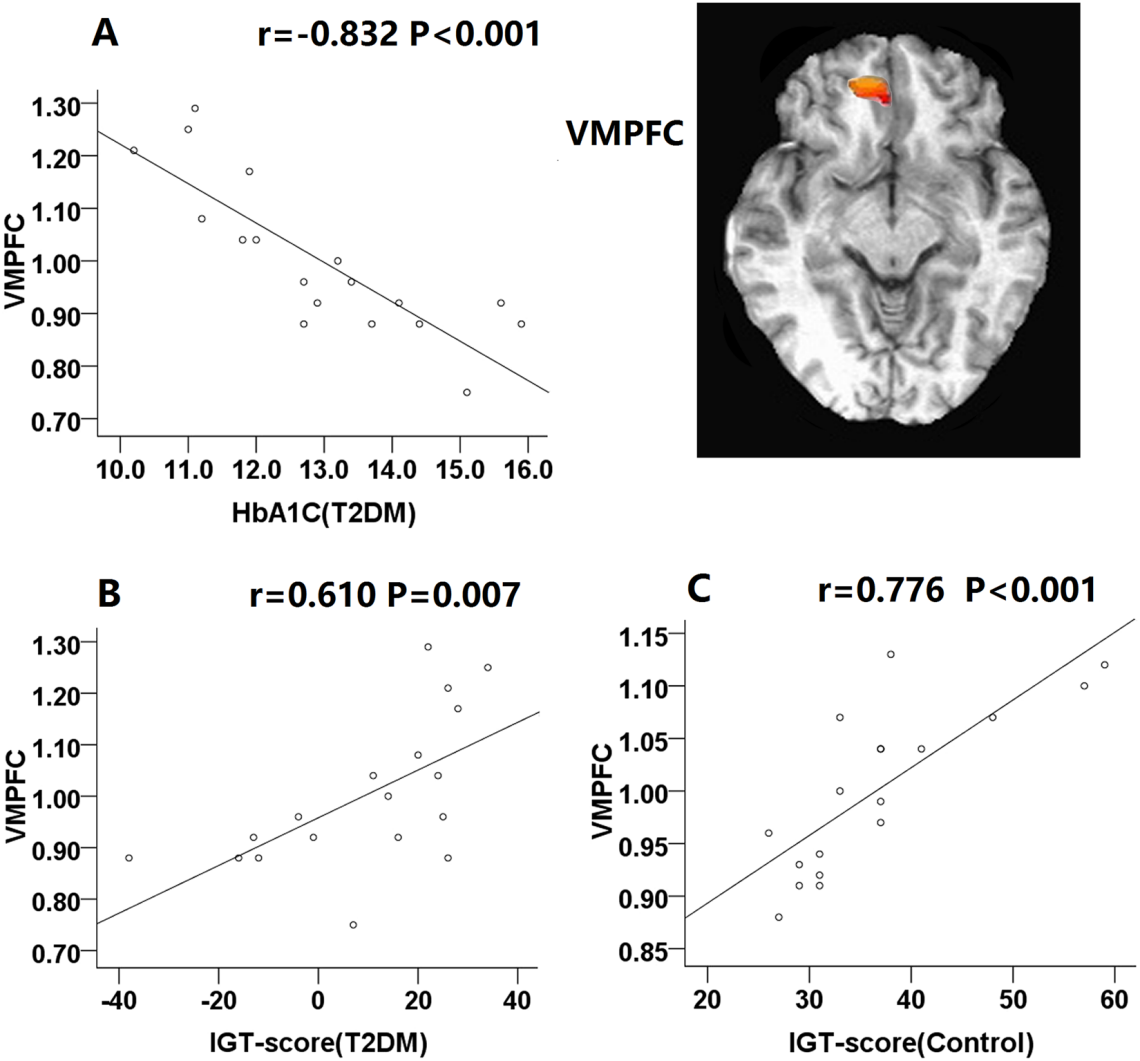
Scatterplots of the ventral medial prefrontal cortex (VMPFC) percent signal change (decision-making - control conditions). Scatterplot (**A**) shows significant negative correlation between HbA1C and VMPFC activation in T2DM patients. Scatterplots (**B**) shows positive correlation between IGT performance and VMPFC activity in the T2DM group. Scatterplots (**C**) shows positive correlation between IGT performance and VMPFC activity in the control group.

**Figure 5:**
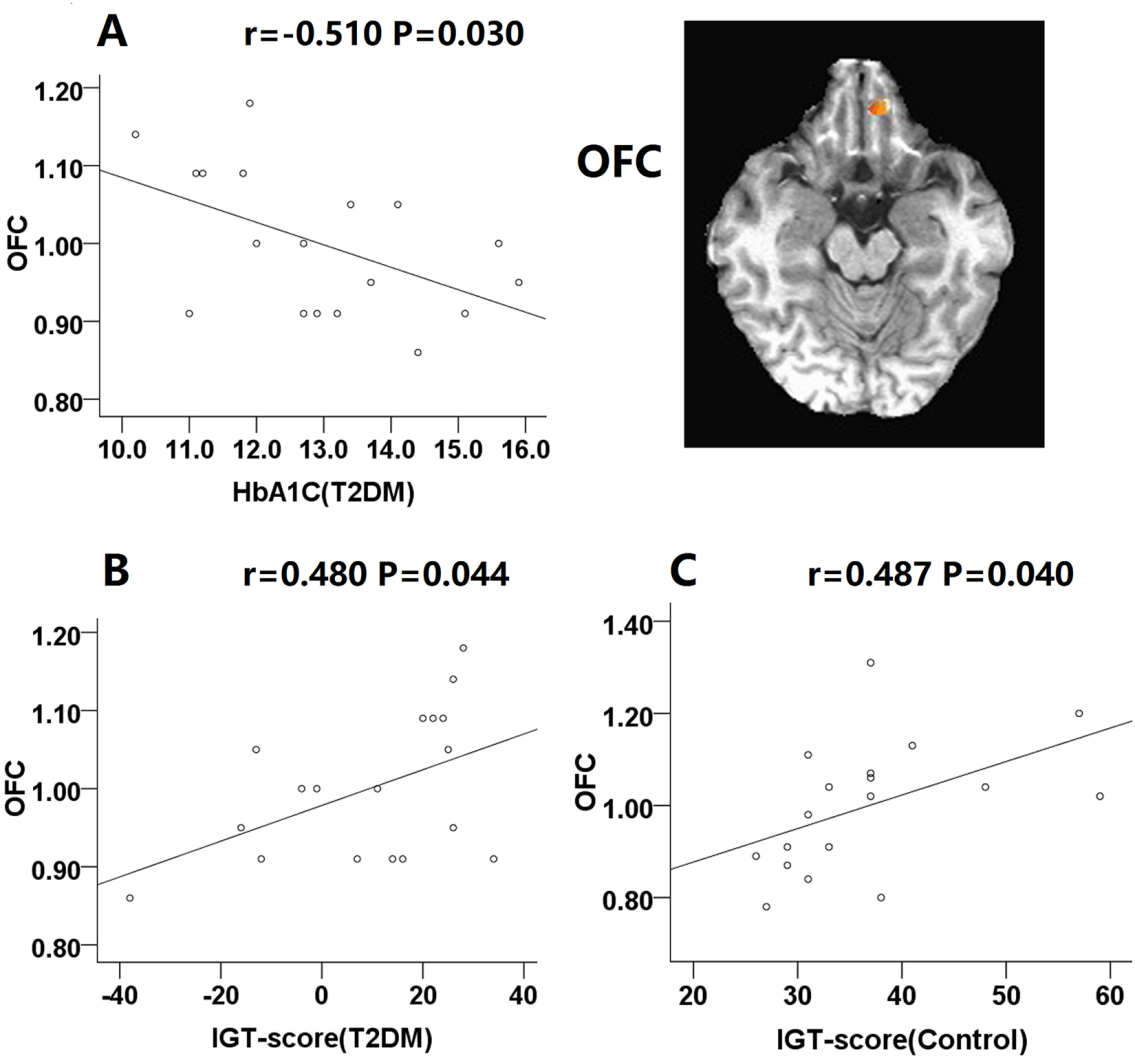
Scatterplots of the orbitofrontal cortex (OFC) percent signal change (decision-making - control conditions). Scatterplot (**A**) shows a negative correlation between HbA1C and OFC activation in T2DM patients. Scatterplots (**B**) shows positive correlation between IGT performance and OFC activity in the T2DM group. Scatterplots (**C**) shows positive correlation between IGT performance and OFC activity in the control group.

In the T2DM group, IGT performance was negatively correlated with HbA1c (*r* = −0.637, P = 0.004). Negative correlations between HbA1c and the brain activations in the VMPFC (*r* = −0.832, P < 0.001) and OFC (*r* = −0.510, P = 0.030) were also revealed in patients, but not in the control group (Figs 4 and 5). No correlations were noted between IGT scores and other clinical data and neuropsychological tests (all p > 0.05).

## Discussion

Our present study provided the first neuropsychological evidence that primary onset middle-aged T2DM patients exhibited altered patterns of brain activation during the Iowa Gambling Task. Block-design fMRI was used to identify the location of impaired brain areas, leading to three main findings. First, T2DM patients exhibited cognitive deficits in the decision-making under ambiguous conditions. Second, they showed less activation in the VMPFC, OFC and ACC, and greater activation in the right DLPFC, insula, posterior cingulate cortex and occipital lobes when compared to controls. A positive correlation existed between IGT performance and activation intensity in the VMPFC and OFC in both groups. Third, T2DM patients with higher HbA1C level (i.e. poor glycemic control) might affect decision-making function, involving both behavioral and brain activations in the OFC and VMPFC. Our current results could help to understand the neural mechanisms of decision-making impairments in T2DM, and provided potential neuroimaging evidence that may be used for early diagnosis and intervention in cognitive decline.

Behaviorally, the results of the present study demonstrated that T2DM patients performed poorly on the IGT, thus confirming previous report on decision making deficits in type 1 diabetes mellitus^15^. No significant deficit was observed in the first two blocks of the IGT, whereas significant impairment in performance in the last three blocks was observed in diabetic individuals as the task progressed. The patients with T2DM more frequently opted for decks with a high immediate reward that were defined as disadvantageous choices, and paid less attention to higher future negative outcomes than controls. These findings were similar to a recent study of T2DM patients in a delay discounting task in which patients preferred smaller, sooner rewards to larger, later ones^37^.

Results showed that diabetic individuals displayed normal but slightly lower cognitive scores in the MMSE test. Then, MOCA total scores were significantly lower for the T2DM group, indicating that T2DM patients possibly have general cognitive impairment. In other investigation studying decision-making, they also have some patients with general cognitive impairment^17, 19^. Nevertheless, it may be more convincing to determine cognitive impairment through a comprehensive neuropsychological battery of standard tests.

Importantly, looking at the IGT performance across blocks in Fig. 1, one can clearly see a learning curve. Although patients’ performance was impaired, T2DM group also showed increasing preference for advantageous decks towards the end of the task, resulting in a positive net score. This is a strong indication that the poorer IGT scores are specifically linked to poor decision-making capacity. Poor IGT scores usually observed in patients with poor memory or early dementia are characterized by random performance (i.e. an absent learning curve, and near zero net scores across all blocks)^11, 12^. Therefore, it seemed that poor decision-making performance in T2DM patients is specifically linked to prefrontal and non-prefrontal neural circuits involved in decision-making, as opposed to other potential non-specific causes (such as poor memory or mild cognitive decline).

The link between general cognitive impairment and deficit in decision-making continues to be debated. Performance in the IGT makes use of diverse cognitive processes, which include executive processing, working memory, cognitive flexibility, attentional resources and planning. Previously it was found that decline in general cognitive function may lead to increased difficulty in decision-making in Alzheimer’s disease patients^12^. However, the majority of IGT studies have failed to observe this association in other populations^19, 38, 39^. More recently, researches show that deficit in decision-making is independent of other cognitive changes occurring during the same period^10, 40^. Consistent with previous studies^19, 38, 39^, we did not find any correlation between impaired decision-making in T2DM group and general cognitive impairment, as measured by the MOCA and MMSE. Further studies are needed to address the potential cognitive factor affecting the decisional process in T2DM group.

Measuring the BOLD signal, in our study, a common network consisting of OFC, VMPFC, DLPFC, ACC, insula and posterior cingulate cortex was activated in the IGT. These are key structures in the influential theoretical neural framework of decision making^8, 9^. Similar decision-making related brain activations have been found in previous positron emission tomography imaging and fMRI studies using the IGT in healthy subjects^9, 41^. In the present study, we further found that the parietal and occipital lobes were also activated.

At the neuroanatomical level, T2DM patients had lower activity than controls in the VMPFC, OFC and ACC, indicating that the altered decision-making capacity in primary onset middle-age T2DM patients is related to the hypofunction of those areas. Similarly, in a recent study of age-related decision-making by fMRI, Halfmann *et al*. suggested older adults with disadvantageous decision patterns showed reduced or absent activation in the VMPFC^42^. Large sample lesion studies of PFC and numerous studies investigating decision making emphasize the importance of the VMPFC and OFC^7, 10, 43, 44^. The VMPFC has been linked to valuation, motivation, reward learning and response inhibition, and is believed to help individuals anticipate and weigh benefits and losses associated with behavioral choices^10, 44, 45^. The OFC is involved in the ability to respond flexibly to situations where one must adjust behavior appropriately in accordance with changing conditions, guiding goal-directed behavior^46, 47^. The OFC activity we observed was within the frontopolar BA10. Studies have found that the BA10 is critically involved in having better foresight–the ability to think of the long-term consequences of one’s behavior and use this information to plan present and future actions^48^. Moreover, the VMPFC and OFC regions are richly interconnected especially via the ventromedial part of the frontal lobe. Hence, the VMPFC and OFC together likely function as a neural network, and it is considered the integrating and operation center of neural mechanisms involved in decision-making. It appears that individuals with VMPFC and OFC dysfunction, which include T2DM patients, may have some difficult in making associations between present emotions and past negative experiences to make an optimal decision-making, despite knowing the adverse consequences.

It is worthy to note that, we found positive correlations between the activation intensity in the VMPFC and OFC and their task performances in both groups, in agreement with earlier fMRI studies of decision-making in the IGT^36, 49^. The participants who performed the IGT successfully exhibited more VMPFC and OFC activation, further supporting previous fMRI study, which suggest that mOFC/VMPFC represent the expected reward value of a choice^50^. The present results imply that reduced activation in these regions are related to performance decline in T2DM. However, less OFC and VMPFC proportional volume have been found in other neurodegenerative diseases such as Parkinson’s disease and Alzheimer’s disease and deficit in decision-making have been related to OFC and VMPFC atrophy in these patients^13, 51^. Future experiments combining fMRI scan and voxel-based morphometry of the VMPFC and OFC in T2DM patients may help to elucidate this relationship.

After controlling for potential confounders, we found that patients with higher HbA1c level showed negative correlation with IGT performance and task-related brain activation in the VMPFC and OFC. It has become apparent that individuals with higher HbA1c level is associated with cognitive decline^52^. Poor glycemic control, defined by high values of HbA1c, is positively associated with the mean blood glucose concentration over previous weeks to months. Hyperglycemia has been implicated in the pathophysiological mechanisms of cognitive decline among T2DM. On the one hand, hyperglycemia will reduce blood flow and suppress blood–brain glucose transport, leading to energy metabolism imbalance and aggravate the cerebral tissue lesion^53^. On the other hand, chronic hyperglycemia might cause accumulation of toxic glucose metabolites, increase oxidative stress and accelerate formation of advanced glycation end-products, which can damage neurons directly and then lead to progressive functional and structural abnormalities in the brain^21^. Overall, our results suggested that poor glycemic control might affect brain activity in the VMPFC and OFC. As mentioned before, the prefrontal cortex plays a crucial role in decision making. Given the evidence that the VMPFC and OFC show significant decreased activation in T2DM patients, it can be speculated that there are concomitant behavior changes associated with alteration in the function of those regions, which could explain the impaired IGT performance. Therefore, the altered brain functional pattern in the present study may help to deepen our understanding of the brain injury mechanisms underlying decision-making related to hyperglycemia.

Besides, Insulin resistance is associated with the occurrence and progression of T2DM. Within the brain, insulin and its signaling pathways not only regulate energy and metabolism balance, but also are associated with cognitive functions^54^. The insulin receptors are distributed throughout the entire brain, especially in cognition-related regions such as the prefrontal cortex and hippocampus^55^. Recent finding also showed that, insulin resistance was negatively correlated with neural activity in the frontal regions in type 2 diabetes^56^. These findings may provide an insight into T2DM-related regional vulnerability, but their exact mechanisms in diabetes still need to be explored.

The T2DM group in current study also exhibited decreased activation in the ACC, a region involved in conflict monitoring and error detection^9, 10, 45^. An earlier fMRI study revealed a conflict -related response in ACC, and the ACC activation increased linearly with incongruity level in the Stroop task^57^. As such, the ACC region may perform a role to predict and avoid risky situations, which are essential for adequate behavioral adaptation and optimum selection strategy. For instance, when a discrepancy occurs between an expected and actual outcome, such processing might assist healthy subjects in avoiding a disadvantageous choice. Reduced activation of the ACC may be a correlate of impaired conflict processing in T2DM patients, which has been suggested as one of the possible causes for poor IGT performance.

Decreased activations can be observed in brain regions as discussed above, abnormally elevated activations were also noted in some brain areas of T2DM individuals including the right DLPFC, insula, posterior cingulate cortex and occipital lobes relative to controls. Increased neuronal activation in these brain regions may be interpreted as an early compensatory recruitment of neuroplasticity that is lost as the disease progresses﻿, which has previously been shown in MCI and Alzheimer’s disease^58, 59^. In general, at the initial onset stage of disease, enhanced brain activity might compensate for the inefficiency of brain regions for decision-making under ambiguous conditions. These compensatory mechanisms are still not clear, and it will be interesting to incorporate more sophisticated measures for structural analysis to advance our understanding on these compensatory responses.

The DLPFC is critical for working memory in decision-making^9^, which would be necessary for active learning of task rules. Based on past findings, the DLPFC is thought to be part of a prefrontal neural network that is significantly affected in T2DM patients, in whom abnormal activation patterns in the DLPFC have been linked to deficits in working memory^33, 34, 60^. This suggests that greater activation in the right DLPFC for working-memory is required to meet the demands of the task. Our study is in line with previous findings showing greater activation in the DLPFC during performance of the working-memory task in newly diagnosed T2DM patients^34^. Besides, neural responses were greater in regions associated with visual attention (occipital and parietal lobes) during decision-making in T2DM patients. These findings indicate patients have heightened the rearrangement and deployment of attentional resources during choices from the decks with their larger reinforcements.

The primary role of the insular cortex is to translate interoceptive signals into what one may subjectively experience as a feeling of desire, anticipation, or urge^61^, which functions as part of an emotion-based system in decision-making. Evidence from a functional neuroimaging study of T2DM has revealed that activity within the insular cortex is implicated in food pictures stimuli^62^. The insula is important for linking response selection process accompanying sensory perception, including those associated with risk taking and punishment. Previous studies show heightened activation in the insular cortex predict more subsequent risky decisions and the activity in the insular cortex also correlates with an individual’s personality trait of urgency^63^. Consistent with these observations, a plausible explanation can be conceived that higher insular activity in our study may correlate with a risky behavior in T2DM patients. This notion is further supported by the increased activation in T2DM patients in the posterior cingulate cortex, an area linked to reward processing and risk preference, and where activity is scaled to the degree of risk when making risky choices^9, 64^.

As we know, BOLD-fMRI remains a useful tool in the indirect assessment of neuronal activity, but BOLD signal may be affected by a variety of factors, such as age, medication, duration, hypertension, hyperlipidemia, brain atrophy and cerebrovascular complications on cognition. To minimize the influences of potential confounding factors, we recruited newly diagnosed middle-aged patients without diabetic complications in a stringent selection and exclusion criteria. Although volumetric measurements of the brain were not performed, subjects with brain atrophy or other abnormalities were ruled out from the current study. In addition, in other investigation studying newly diagnosed middle-aged T2DM patients, a voxel-based morphometry analysis on the anatomical imaging data did not find significant differences in grey matter volume at neither global nor regional level^34^. Therefore, we consider that the differences in activation we observed between the groups are unlikely to be confound by brain atrophy, especially the middle-age primary onset patients. Multimodal imaging to assess structurally and functional changes in brains of T2DM patients may be used for future research to validate our hypothesis.

Additionally, the possible vascular lesions in T2DM patients should not be ignored. Duarte *et al*. showed a distinct hemodynamic response function between T2DM patients (average age 59.73 years, average duration of disease 12.37 years) and controls, then brain activation can be influenced by a changed vascular reactivity and impaired neurovascular coupling in patients with diabetes^65^. Although we did not have detailed vascular health status within our subjects, we excluded participants with a history of ischemic heart disease, peripheral vascular disease or other microvascular complications. Besides, no evidence of cerebral infarcts, cerebral microbleeds or white matter hyperintensity was identified in the current study, and no differences between our groups in age, systolic/diastolic blood pressure, triglyceride, total cholesterol levels or BMI (see Table 1). Moreover, to mitigate the effect of possible vascular lesions, we subtracted BOLD responses in non-gambling control conditions from those of decision-making conditions to objectify activations associated with the integration of information about a complex decision. We consider that the effect of decision-making on the BOLD response within a certain range is not qualitatively altered by noncognitive factors, and perhaps it is a feasible way to check the existence of functional differences in decision-making. However, given the current study design, we were not able to discard the relative contribution of potential cerebrovascular lesions, which should be explored in future studies with more specific MRI techniques.

While the results may give insight into the effects of hyperglycemia on decision-making cognitive processes, several limitations of our study deserve mention. The current study is limited by its relatively small sample size. Therefore, our results should be interpreted cautiously and need further validation using a larger sample size. Secondly, we acknowledge that by focusing neurophysiological analyses on the activated regions induced by the block-design of the IGT addresses only one aspect of IGT performance. Using IGT in an event-related fMRI experiment will allow us to extract relative timing information on the onset of activity in different neural substrates and disentangle various aspects of decision-making, such as anticipation, choose and feedback. An event-related design of the IGT task could be applied for further study. Thirdly, only global assessment of cognitive impairment was performed. By incorporating more detailed tests of cognitive function (e.g. Wisconsin card sorting test, backwards digit-span test, Stroop test), future studies are needed to investigate the interaction between decision-making and other cognitive functions.

To the best of our knowledge, this is the first study that seeks to assess the associations between T2DM, decision-making, and BOLD-fMRI while minimizing the confounding influences of diabetes-associated factors. The patterns of brain activity during the IGT suggest that portions of the neural systems underlying decision-making processes are altered in T2DM patients. In other words, our study shows that newly diagnosed middle-aged T2DM patients may suffer from weaknesses in their prefrontal cortex functions that lead to poorer decision-making under ambiguous conditions, at least as assessed by the IGT. These results also imply an adverse impact of poor glycemic control on decision-making and functional abnormalities in prefrontal neural networks. Our findings are of great clinical significance, because early detection of brain function abnormalities in middle age may allow for proactive therapies that can prevent or reduce future cognitive decline. Further studies are needed to replicate these results and to evaluate the clinical value of brain imaging methods in the prediction of disease progression in these patients.

## Materials and Methods

### Participants

The Medical Ethics Committee of Shantou University Medical College approved the study. Informed consent forms were signed by all subjects after a full description of the MRI examination in the study was provided. The methods and experimental procedures were carried out in accordance with the approved guidelines.

All patients had been newly diagnosed with T2DM, without any treatments, such as food control and medication. According to the American Diabetes Association (ADA) recommendation and prior diagnostic criteria, the T2DM group inclusion criteria consisted of the following: (1) age 35–55 years; (2) symptoms of diabetes, random plasma glucose ≥11. 1 mmol/L; (3) fasting plasma glucose ≥7.0 mmol/L; (4) 2-hour plasma glucose ≥11.1 mmol/L during an oral glucose tolerance test; and (5) MMSE scores ≥24, MOCA scores ≥19, HAMD scores <8. The exclusion criteria were: (1) a history of type 2 diabetes mellitus; (2) evidence of abnormalities in the brain (e.g. brain atrophy, white matter hyperintensity, cerebral infarcts or cerebral microbleeds); (3) any other types of systemic diseases impairing cognitive functioning; (4) a history of neurological or psychiatric disease; (5) a history of clinically diagnosed stroke or severe, uncontrolled cardiovascular disease; (6) a history of alcohol or substance misuse; or (7) contraindication for MRI scanning. High-resolution T1- and T2-weighted axial scans covering the whole brain of each participant were evaluated to exclude the presence of atrophy or other structural changes (e.g. white matter hyperintensity, cerebral infarcts or cerebral microbleeds). Three professional neuroradiologists blinded to the group allocations performed the analyzation separately. Consensus was obtained through discussion between the three experts.

After quality control, eighteen T2DM patients were identified by an endocrinologist from the department of Endocrinology, the First Affiliated Hospital of Shantou University and Shantou Chaonan Minsheng Hospital from February 2015 to June 2016. The control sample consisted of 18 demographically matched healthy volunteers, who met the same exclusion criteria as the patients. All subjects were right-handed, and had normal or correct-to-normal vision.

### Clinical data and neuropsychological assessment

Clinical diagnoses were conducted on all subjects after a baseline medical screening, a neuropsychological battery, and a laboratory examination. Body mass index was calculated as weight in kilograms divided by the square of the height. Venous blood samples were collected at 8 A.M. after an overnight fast of at least 10 hours. Fasting blood glucose, glycosylated hemoglobin, triglyceride, and total cholesterol levels were assessed.

The MMSE and MOCA tests were administrated to all subjects to evaluate the individual’s general cognitive status by two experienced neuropsychiatrists. Both the MMSE and the MOCA exams used a scale of 0–30 points. Those scoring 26 points or greater were considered as having normal cognitive function, and <26 points as possibly having mild cognitive dysfunction. MMSE Scores <23 or MOCA Scores <19 were used for excluding possible dementia. Because depression is important in research focused on cognitive function and is more common in T2DM patients, the Hamilton Depression Scale was also administered.

### Iowa Gambling Task Modified for fMRI

As described in previous studies^7^, all participants completed an fMRI-adapted version of the IGT that included five blocks of decision-making conditions interleaved with five blocks of non-gambling control conditions. Each decision-making block consisted of 20 card selections. During each trial, subjects had three seconds to make the selection, followed by a three-second feedback screen during which they learned the amount won or lost for that trial. The task was programmed and presented using E-Prime Software.

The decision-making conditions involved a computerized task in which the participant saw four decks of cards on a screen. For each trial, the participant selected a card from any of the four decks. Each card selection yielded a gain, but it could also yield a loss. The amounts won and lost were then displayed, and the display also included the overall cumulative payoff, which was updated with each trial. The reward/penalty schedules were predetermined: Deck A and B yielded high immediate rewards, but carried a risk of much higher long-term penalties, that resulted in a total loss in the long run (disadvantageous decks); Decks C and D yielded low immediate rewards, but smaller long-term penalties, that resulted in a long-term gain (advantageous decks).

The visual stimuli and motor demands for non-gambling control conditions were as identical to the decision-making conditions presentation as possible: four card decks were presented, just as in the decision-making conditions. Subjects were instructed to select a card from a specific deck (e.g. “Select Deck B”). Thus, the non-gambling control conditions contained all the visual and feedback characteristics of the experimental tasks, but without the requirement to make a complex decision, as required in the decision-making conditions.

### MR data acquisition

Imaging was carried out on a 1.5 T General Electric MR scanner with a quadrature coil-acquired signal. Cross-sectional images were acquired to correct for the localization of axial anatomical images. All image acquisitions were performed by experienced neuroradiologists. For anatomical reference, we acquired a higher resolution coplanar T1-weighted series, with repetition time/echo time [TR/TE] = 505/14 ms, flip angle = 90°, 20 contiguous slices, with slice thickness = 6 mm (no gap), field of view[FOV] = 230 × 230 mm, matrix = 256 × 256. Functional images were acquired at the same location as the anatomical slices by using a gradient-recall echo, echo-planar imaging sequence, acquiring 20 slices (6 mm thick, no gap), TR/TE = 3000/45 ms, flip angle = 90°, FOV = 230 × 230 mm, matrix = 64 × 64. One functional run was collected for each participant. Finally, a high-quality three-dimensional (3-D) image was collected with a fast low-angle radiofrequency pulse sequence, with TR/TE = 30/3.0 ms, flip angle = 30°, FOV = 230 × 230 mm, matrix = 256 × 256, slice thickness = 1.3 mm, no gap, and 120 slices were acquired.

### Data analysis

#### Statistical analysis

Clinical and neuropsychological data were tested for normality. Comparisons were performed for the two groups using independent t-tests for continuous variables and chi-square tests for categorical variables. The IGT net score was calculated as the difference between the number of choices from two advantageous (net positive reward outcome, C + D) minus two disadvantageous (net negative reward outcome, A + B) decks, a measure of how well participants learned the expected value of the decks to guide their choice. Positive scores for net score indicated an advantageous strategy (overall gain), whereas negative scores represented a dominance of disadvantageous deck choices (overall loss). A 2 (group) × 5 (block) repeated measures ANOVA on the net score was used to assess the learning effect, with the block as the within-subject factor and the group (patients vs. controls) as the between-subject factor.

To determine if brain activity was related to task performance, we carried out correlation analyses between the total net scores and brain activations during decision-making. Given that HbA1c was assayed as an estimate for glycemic control, the potential association between HbA1c and task-related BOLD responses, as well as the IGT performance were tested with correlational analysis. Correlational analysis was also calculated between IGT performance and other clinical data and neuropsychological measures. Correlations involving data from all subjects were analyzed using Pearson’s correlation coefficient.

Statistical analyses were conducted using the Statistic Package for Social Science (SPSS) version 19.0 for Windows (IBM, Armonk, NY, USA). A value of P < 0.05 was considered statistically significant.

#### fMRI image analysis

Firstly, the original data were converted using MRI Convert software (http://lcni.uoregon.edu/). MRI data were then analyzed using AFNI (Analysis of Functional NeuroImages, a set of C programs for processing, analyzing and displaying fMRI data. http://afni.nimh.nih.gov/afni/), including data preprocessing, statistical analysis and result display. In data preprocessing, all functional datasets were pre-processed to remove any linear drift, to correct for motion, be normalized to the stereotaxic coordinates of Talairach and Tournoux (1988), and spatially smoothed with a Gaussian full width half maximum 6.0 mm filter. Any scan in which the head motion was larger than 2 mm or rotation larger than 10° was excluded from subsequent analysis. All participants did not have obvious structural abnormalities in the brain, and all functional data were kept for further image analysis.

Correlational analyses based on the direct contrast between the decision-making and control conditions were carried out to generate the activation map for each group (p < 0.05, cluster size >20 voxels). These activation maps were used to locate the regions of interest (ROIs). For each subject, a correlational analysis was performed on the functional data to generate two activation maps and a combined activation map by the logical ‘OR’ of these maps (p < 0.05, cluster size >20 voxels). The amplitudes of the average BOLD responses were calculated for each subject (decision making - control conditions).

### Data Availability

The datasets generated during and/or analysed during the current study are not publicly available due to our study of middle-age T2DM patients in going on, but are available from the corresponding author on reasonable request.
